# Identification and Validation of the miR156 Family Involved in Drought Responses and Tolerance in Tea Plants (*Camellia sinensis* (L.) O. Kuntze)

**DOI:** 10.3390/plants13020201

**Published:** 2024-01-11

**Authors:** Shengjing Wen, Chengzhe Zhou, Caiyun Tian, Niannian Yang, Cheng Zhang, Anru Zheng, Yixing Chen, Zhongxiong Lai, Yuqiong Guo

**Affiliations:** 1 College of Horticulture, Fujian Agriculture and Forestry University, Fuzhou 350002, China; m18816476976@163.com (S.W.); chengzhechou@foxmail.com (C.Z.); cytian1997@foxmail.com (C.T.); niannian124@foxmail.com (N.Y.); zhangcheng9742@foxmail.com (C.Z.); zar527@foxmail.com (A.Z.); cyxing2001@163.com (Y.C.); laizx01@163.com (Z.L.); 2 Institute of Horticultural Biotechnology, Fujian Agriculture and Forestry University, Fuzhou 350002, China; 3 Anxi College of Tea Science (College of Digital Economy), Fujian Agriculture and Forestry University, Quanzhou 362400, China

**Keywords:** tea plant, miR156s, drought stress, SQUAMOSA promoter binding protein-like

## Abstract

The microRNA156 (miR156) family, one of the first miRNA families discovered in plants, plays various important roles in plant growth and resistance to various abiotic stresses. Previously, miR156s were shown to respond to drought stress, but miR156s in tea plants (*Camellia sinensis* (L.) O. Kuntze) have not been comprehensively identified and analyzed. Herein, we identify 47 mature sequences and 28 precursor sequences in tea plants. Our evolutionary analysis and multiple sequence alignment revealed that csn-miR156s were highly conserved during evolution and that the rates of the csn-miR156 members’ evolution were different. The precursor sequences formed typical and stable stem–loop structures. The prediction of *cis*-acting elements in the *CsMIR156s* promoter region showed that the *CsMIR156s* had diverse *cis*-acting elements; of these, 12 *CsMIR156s* were found to be drought-responsive elements. The results of reverse transcription quantitative PCR (RT-qPCR) testing showed that csn-miR156 family members respond to drought and demonstrate different expression patterns under the conditions of drought stress. This suggests that csn-miR156 family members may be significantly involved in the response of tea plants to drought stress. Csn-miR156f-2-5p knockdown significantly reduced the *Fv/Fm* value and chlorophyll content and led to the accumulation of more-reactive oxygen species and proline compared with the control. The results of target gene prediction showed that csn-miR156f-2-5p targeted SQUAMOSA promoter binding protein-like (*SPL*) genes. Further analyses showed that *CsSPL14* was targeted by csn-miR156f-2-5p, as confirmed through RT-qPCR, 5′ RLM-RACE, and antisense oligonucleotide validation. Our results demonstrate that csn-miR156f-2-5p and *CsSPL14* are involved in drought response and represent a new strategy for increasing drought tolerance via the breeding of tea plants.

## 1. Introduction

Drought stress is a devastating and unavoidable reality that is becoming increasingly prevalent in most parts of the world, and research data show that flash droughts occur frequently worldwide [1,2]. Drought not only causes serious agricultural production losses but also ecological damage, desertification, and soil erosion, among other things. As a result, drought has been recognized as an urgent global and environmental issue [3,4]. The tea plant (*Camellia sinensis* (L.) O. Kuntze) is an economically important crop grown all over the world. Drought stress is one of the most prominent natural challenges threatening major tea-producing areas in China; it remarkably affects the growth and development, yield, and quality of tea plants. Tea plants are sessile organisms that cannot escape drought when it occurs. Thus, to withstand drought stress, like other plants, tea plants display a series of interconnected response and defense mechanisms to safeguard their survival, which involve multiple processes. These may be morphological, physiological, or related to water stress sensing, stress signal transduction, and related gene expression regulation [5]. Recent studies have shown that (Z)-3-hexanol and eugenol can enhance the drought tolerance of tea plants by mediating glucosylation [6]. An exogenous application of 24-epibrassinolide can improve the drought resistance of tea plants by promoting the expression of genes involved in the biosynthesis of galactinol and abscisic acid [7]. In addition, several genes such as *CsUGT71A59*, *CsGSTU8*, and *CsSnRK2.5* have shown the ability to enhance drought tolerance by regulating the accumulation of reactive oxygen species and altering abscisic acid homeostasis [8,9,10]. However, the specifics of the tea plant’s drought resistance mechanism are still unclear. Therefore, it is necessary to study said drought resistance mechanism and thereby improve the drought tolerance of tea plants.

MicroRNAs (miRNAs) are a class of 20–24-nucleotide (nt) endogenous non-coding RNAs in eukaryotes that regulate gene expression at the transcriptional, post-transcriptional, or translational level through complementary binding with target genes. As an important component of gene expression regulation, miRNAs play an important role in plant growth, development, and response to stress through the precise regulation of corresponding target genes [11,12]. Recently, the strategy of fine-tuning miRNAs based on the regulation of target gene transcription has been highlighted as an effective biotechnological method of improving tolerance to abiotic or biotic stresses in economically vital crops. Several studies have confirmed that many miRNAs, such as miR156 [13,14], miR166 [15], miR319 [16], and miR408 [17,18], can improve plant stress resistance to biotic or abiotic stresses by overexpressing or knocking out miRNAs. In apples, *MIR156a* overexpression weakens salt resistance [19]. The knock-down of miR166 can enhance abiotic stress resistance [20]. The overexpression of *MIR319b* and miR319 in the target simulated form (MIM319) demonstrated that miR319 positively regulates salt tolerance in switchgrass [21]. The drought resistance of Os-miR408 transgenic plants can be improved through changes in their leaf morphology [22]. miR156s are a family of small RNAs that were one of the first miRNAs discovered in Arabidopsis; they are highly conserved in plant species. In general, miR156s regulate the expression of the SQUAMOSA promoter binding protein-like (*SPL*) gene family through transcriptional cleavage or translation inhibition, thus inducing resistance to drought stress. *SPLs* are a family of plant-specific transcription factors with highly conserved SBP domains that play an essential role in plant growth and development and response to stress [14,23,24]. A recent study showed that the miR156-mediated silencing of *SPLs* improved drought stress resilience and promoted leaf gas exchange and abscisic acid sensitivity [25,26]. In alfalfa, miR156 was shown to improve drought resistance by downregulating *SPL13* expression; in the same study, the overexpression of miR156 led to stronger drought resistance [13]. Therefore, we used an analysis of the csn-miR156 family as the starting point of our study, progressing to explore the regulating effect of csn-miR156s on drought resistance.

In this study, 47 mature sequences and 28 precursor sequences in tea plants were identified based on published small-RNA sequencing results. Herein, we will provide a detailed overview of the evolution of miR156s and pre-miR156s in tea plants. We detected the expression level of csn-miR156s while being affected by different degrees of drought using RT-qPCR; then, csn-miR156f-2-5p was screened and knocked down. Compared to the control, the csn-miR156f-2-5p-knockdown tea plants showed lower tolerance to drought stress. In order to further explore the drought resistance mechanism of csn-miR156f-2-5p, we also predicted and analyzed the csn-miR156f-2-5p targets. The relationship between csn-miR156f-2-5p and its target genes was verified using reverse transcription quantitative PCR (RT-qPCR) and 5′RACE, followed by antisense oligonucleotide (AsODN) treatment in tea plants. To summarize, we explored the mechanisms mediated by the miR156-SPL regulatory module during drought in tea plants to provide a reference for breeding drought resistance in tea plants.

## 2. Results

### 2.1. Identification and Analysis of miR156 in Plants

**Figure 1:**
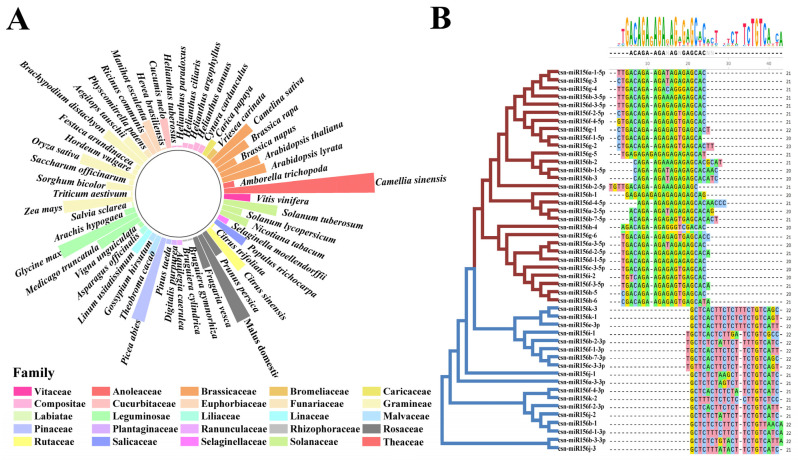
Statistics of plants’ miR156 sequences alongside multiple sequence alignment of csn-miR156s. (**A**) Numeric distribution of miR156s in 55 plant species; the height of the column indicates their quantity. (**B**) Classification and multiple sequence alignment of miR156 family members in *Camellia sinensis*.

To understand the distribution of the miR156 family in plants, a total of 447 mature sequences of miR156 from 54 species were downloaded from miRbase, and 47 csn-miR156 mature sequences were identified from published small-RNA sequencing results [27,28,29,30,31]. The miR156s were widely distributed in angiosperms, gymnosperms, and mosses, and the number of miR156s in angiosperms was the largest. The number of miR156 members in each species ranged from 1 to 47 (Figure 1A). To further investigate the evolutionary relationship between miR156s in the plants, we used *Physcomitrella patens* (3), *Selaginella moellendorffii* (4), *Oryza Sativa* (18), *Glycine max* (28), *Vitis vinifera* (9), *Arabidopsis thaliana* (13), *Medicago truncatula* (15), *Nicotiana tabacum* (10), *Brassica napus* (7), *Picea abies* (27), and *Camellia sinensis* (47) to construct a phylogenetic tree (Figure S1). The phylogenetic tree was divided into three clades. All the miR156s derived from the 5′ end of the precursor, such as csn-miR156a-1-5p, osa-miR156l-5p, mtr-miR156i-5p, ath-miR156c-5p, and so on, belonged to clade I and clade II. Clade III consisted of miR156s derived from the 3′ end of the precursor and csn-miR156i-1, csn-miR156j-1/2/3, and csn-miR156k-2.

Alignment of the mature sequences of the miR156 family of the above 10 plants showed that miR156s were roughly divided into two groups based on whether they were derived from the 5′ or 3′ arms of the precursor (Figure S2). The results showed that most miR156 family members of different species were 21 nt, and a few were 20 nt or 22 nt. Except for a few base mutations, deletions, or insertions in the 12th, 15th, and 16th bases, the rest of the bases are highly conserved. Further comparison of the mature sequence of miR156 in tea plants showed that the csn-miR156s were divided into two types: the 5′-end sequence and the 3′-end sequence (Figure 1B). Except for the 1st, 12th, and 15th base differences at the 5′ end and the 5th, 13th, and 19th base differences at the 3′ end, the bases of the csn-miR156s were conserved.

### 2.2. Sequence Analysis and Chromosomal Localization of csn-MIR156s in the Tea Plant Genome

**Figure 2:**
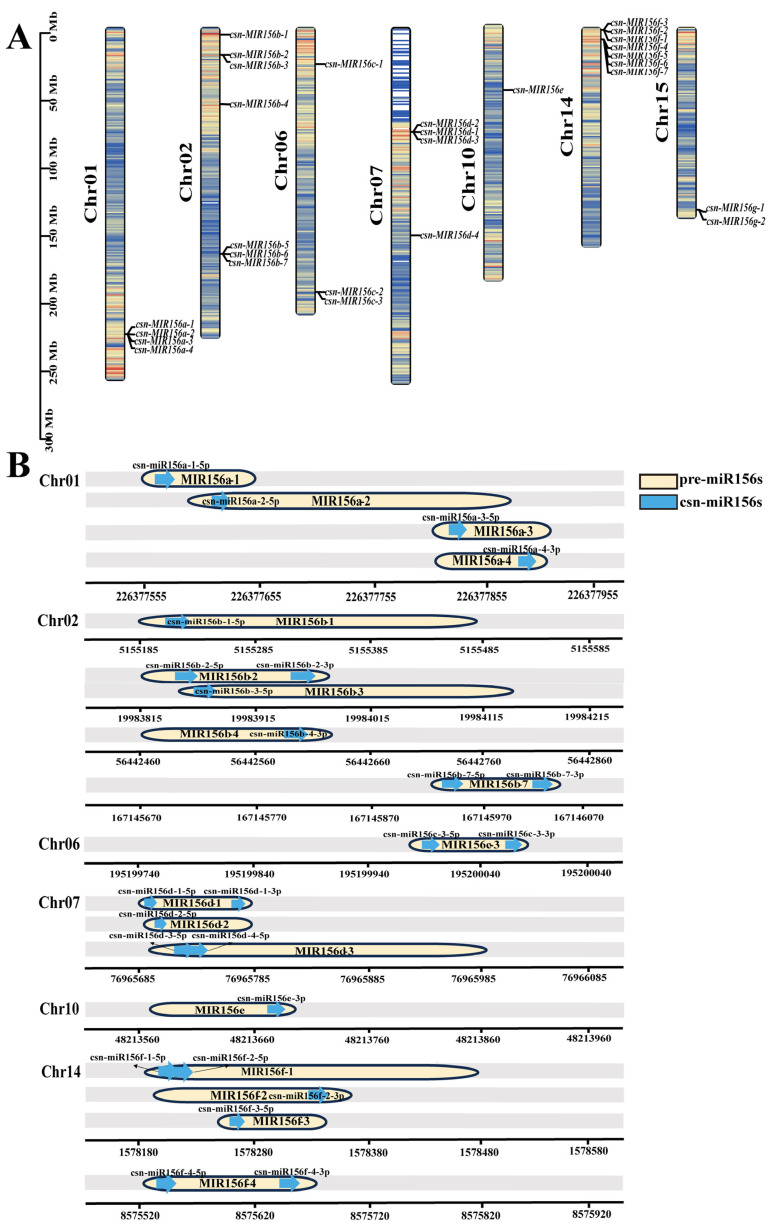
Location of the csn-MIR156 family in the ‘Tieguanyin’ genome. (**A**) Location of csn-miR156s’ precursor sequences on chromosomes. The different colors in the chromosomes represent gene density. (**B**) The position of mature csn-miR156s on precursors. The blue arrow represents the mature sequence, and the yellow capsule represents the precursor sequence.

Based on published small-RNA sequencing results, 28 precursor sequences of the miR156 family were identified in the tea plants. The identified pre-miR156s were within 300 nt, meaning they were within the normal range of plant miRNA precursor length, and the sequences were conserved at the 5′ and 3′ ends (Figure S3). To determine the chromosomal locations of csn-miR156 family members, we aligned the precursor sequences of csn-miR156s (csn-MIR156s) with the ‘Tieguanyin’ genome (Figure 2A). The csn-MIR156s were located on chr01 (csn-MIR156a), chr02 (csn-MIR156b), chr06 (csn-MIR156c), chr07 (csn-MIR156d), chr10 (csn-MIR156e), chr14 (csn-MIR156f), and chr15 (csn-MIR156g). Csn-miR156a/b/c/d/e/f were named according to the position of the mature sequence on their corresponding precursor (Figure 2B). Among them, csn-miR156f-1-5p and csn-miR156f-2-5p were likely derived from csn-MIR156f-1 on chr14, while csn-miR156g/h/i/j/k did not correspond to the 28 precursor sequences.

### 2.3. Predicted Secondary Structure Analysis and Phylogenetic Analysis of csn-MIR156s in the Tea Plant Genome

In order to understand the secondary structure of csn-MIR156s, the RNAfold web server (http://rna.tbi.univie.ac.at/cgi-bin/RNAWebSuite/RNAfold.cgi; accessed on 25 July 2023) was used, with default parameters, to analyze the secondary structures of the csn-MIR156s. The results showed that almost all the csn-MIR156s formed typical and stable structures with a stem–loop hairpin (Figure S4). In addition, we also labeled the location of the mature sequence on the secondary structure of the precursor and found that the csn-MIR156s’ secondary structures were highly stable in the region (arm) of the mature miRNA sequences, while the rest of the sequences (loop) were more unstable. The minimum folding free energy of the csn-MIR156s varied considerably, ranging from −95.60 to −13.40 kcal/mol. The minimum folding free energy was proportional to the sequence length of the csn-MIR156s.

**Figure 3:**
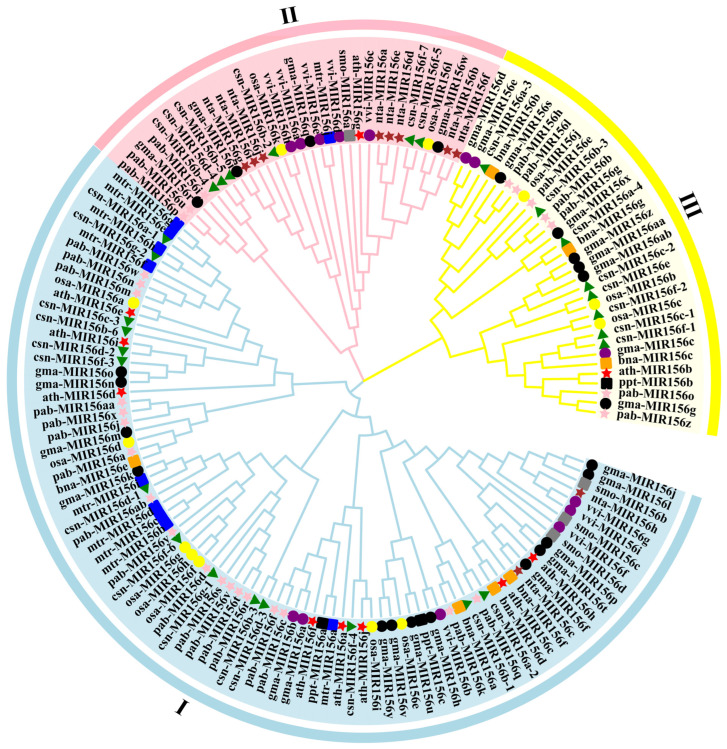
Phylogenetic tree of plants’ MIR156 sequences. vvi. *Vitis vinifera*; smo. *Selaginella moellendorffii*; osa. *Oryza sativa*; csn. *Camellia sinensis*; ath. *Arabidopsis thaliana*; gma. *Glycine max*; mtr. *Medicago truncatula*; nta. *Nicotiana tabacum*; bna. *Brassica napus*; pab. *Picea abies*; ppt. *Physcomitrella patens*.

To further examine the evolutionary relationship among the 28 csn-MIR156s, we used MEGA 10.0 software to analyze the evolution of the pre-miR156 sequences from *P. patens* (3), *S. moellendorffii* (4), *O. sativa* (12), *V. vinifera* (nine), *A. thaliana* (10), *G. max* (28), *M. truncatula* (10), *N. tabacum* (10), *C. sinensis* (28), *B. napus* (seven), and *P. abies* (27). The MIR156s were mainly divided into three branches. Specifically, 18 pab-MIR156s (pabMIR156a/c/f/d/j/k/m/n/q/v/r/s/t/w/x/y/aa/ab/), 15 gam-MIR156 (gam-MIR156a/b/f/h/j/k/l/m/n/o/p/t/u/v/y), 14 csn-MIR156 (csn-MIR156a-1/a-2/b-1/b-3/b-6/c-3/d-1/d-2/d-3/f-3/f-4/f-6/g-1/g-2), 7 osa-MIR156 (osa-MIR156a/d/e/f/g/h/i), 4 vvi-MIR156s (vvi-MIR156b/f/g/i), 2 nta-MIR156 (nta-MIR156c/h), 8 mtr-MIR156 (mtr-MIR156b/c/d/e/f/g/h/i), 8 ath-MIR156 (ath-MIR156a/c/d/e/f/h/i/j), 4 bna-MIR156 (bna-MIR156a/d/e/f), and 2 ppt-MIR156 (ppt-MIR156a/c) members were clustered into a broad branch. The second branch consisted of six csn-MIR156s (csn-MIR156b-2/b-4/b-5/d-4/f-5/f-7), three pab-MIR156s (pab-MIR156i/p/u), four gam-MIR156s (gam-MIR156i/q/r/w), five vvi-MIR156s (vvi-MIR156a/c/d/e/h), two osa-MIR156s (osa-MIR156k/l), eight nta-MIR156s (nta-MIR156a/b/d/e/f/g/i/j), one mtr-MIR156 (mtr-MIR156j), and one ath-MIR156 (ath-MIR156g). Eight csn-MIR156s (csn-MIR156a-3/a-4/b-4/c-1/c-2/e/f-1/f-2), six pab-MIR156s (pab-MIR156b/e/g/h/l/o/z), nine gam-MIR156s, three osa-MIR156s, one ath-MIR156s, three bna-MIR156s, and one ppt-MIR156 (ppt-MIR156b) were grouped into the third branch (Figure 3).

Further analysis revealed that the 28 miR156 precursors of the tea plant were present in three branches, as well as in *P. abies*, *A. thaliana*, *M. truncatula*, and *G. max*. Studies have shown that the evolutionary distance of miRNA families is not closely related to the genetic relationship between species [32,33,34]. In this study, members of csn-MIR156 and the MIR156s of monocotyledons, dicotyledons, gymnosperms, and ferns were widely distributed on the phylogenetic tree (Figure 3), suggesting that the csn-MIR156 family is a conserved and ancient miRNA family.

### 2.4. Analysis of Cis-Acting Elements in CsMIR156s Promoter Regions

**Figure 4:**
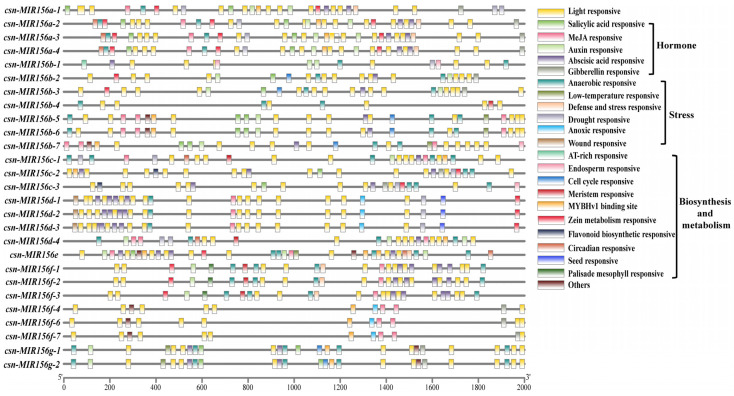
Promoter analysis of *CsMIR156* genes in *C. sinensis*.

Transcription initiation is a vital stage of gene expression, and promoters are an important regulatory component of gene transcription initiation. The identification and analysis of the *cis*-acting elements of miRNA promoters will help to further our understanding of the transcriptional activation mechanism of miRNA, as well as its role in the overall regulatory network [35]. Therefore, to identify the function of *CsMIR156s*, 2000 bp upstream regions of *CsMIR156s* in tea plants were used as putative promoter regions for the prediction of *cis*-acting elements. In 28 *CsmiR156s* promoters, in addition to the core promoter element TATA-box and common CAAT-box elements, a total of 52 specific promoter *cis*-acting elements were identified. (Table S1), which were divided into the following five categories: light-responsive, hormone-responsive, stress-responsive, biosynthesis- and metabolism-related *cis*-acting elements, and others (A-box, 3-AF3 binding sites, 60K protein binding sites, and AT-rich sequences) (Figure 4). Among them, the ‘light-responsive’ (44.7%) category was shared among the 28 *csn-MIR156s*, and six stress-response elements, namely, ‘anaerobic-responsive element’ (63.6%), ‘drought-responsive element’ (15.0%), ‘defense and stress-responsive element’ (7.5%), ‘low-temperature-responsive element’ (5.6%), ‘anoxic-responsive element’ (5.6%), and ‘wound-responsive element’ (2.8%), were identified. A total of 12 *CsMIR156s* (*CsMIR156a-1/a-2/a-3/a-4/b-3/c-1/c-2/c-3/d-1/d-2/d-3*) were identified as possessing drought-responsive elements (MBS).

### 2.5. Expression Analysis of csn-miR156s under Drought Stress

**Figure 5:**
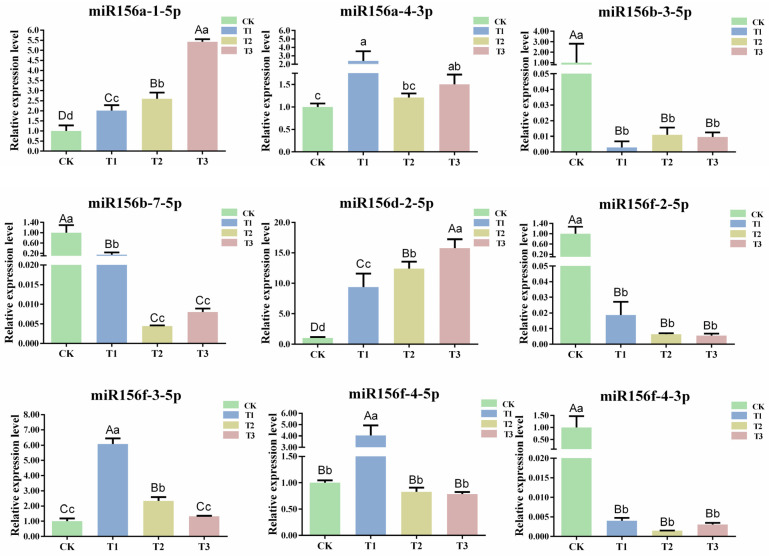
Expression patterns of csn-miR156s under different degrees of drought. CK: normal water supply; T1: mild drought stress, T2: moderate drought stress, and T3: severe drought stress. Data are the means of three independent replicates ± standard deviation (SD). Different lowercase letters indicate significant differences (*p* < 0.05); different uppercase letters indicate highly significant differences (*p* < 0.01).

The length of miRNAs that guide AGO1 to cleave their target mRNAs in plants is generally 21 nt [12]. To explore the expression pattern of csn-miR156s under different drought degrees, nine 21 nt csn-miR156s that could localize on precursors (csn-miR156a-1-5p, csn-miR156a-4-3p, csn-miR156b-3-5p, csn-miR156b-7-5p, csn-miR156d-2-5p, csn-miR156f-2-5p, csn-miR156f-3-5p, csn-miR156f-4-5p, and csn-miR156f-4-3p) were chosen to detect expression patterns under drought stress via RT-qPCR. The expression patterns of the csn-miR156 members displayed a different trend in response to different degrees of drought. Specifically, the expression levels of miR156a-1-5p, miR156a-4-3p, miR156d-2-5p, and miR156f-3-5p showed a similar tendency. Their expression levels were upregulated compared with CK under T1, T2, and T3. The expression level of miR156f-4-5p increased significantly at T1 but decreased insignificantly at T2 and T3. Concurrently, the expression levels of miR156b-3-5p, miR156b-7-5p, miR156f-4-3p, and miR156f-2-5p decreased under different drought degrees compared with CK, and that of miR156f-2-5p declined continuously (Figure 5). Various studies have revealed that the expression levels of miR156s (mdmiR156a and ath-miR156) are continuously downregulated under abiotic stress, and miR156f-2-5p showed the same trend [19,36]. Therefore, miR156f-2-5p was selected for further analysis.

### 2.6. csn-miR156f-2-5p Participates in Drought Stress Response in Tea Plants

**Figure 6:**
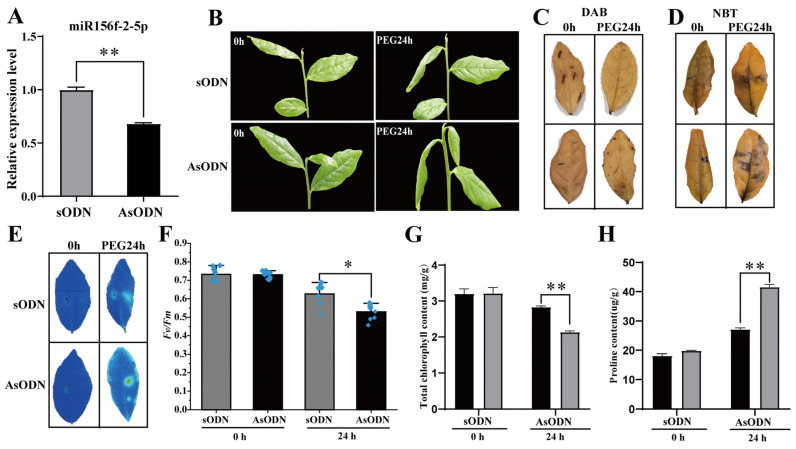
Csn-miR156f-2-5p plays a role in the drought tolerance of tea plants. (**A**) Expression levels of csn-miR156f-2-5p control (sODN) and csn-miR156f-2-5p-knockdown (AsODN) tea plants. (**B**) Phenotypes of the control (sODN) and csn-miR156f-2-5p-knockdown (AsODN) tea plants after 0 h and 24 h of being subjected to drought conditions. (**C**,**D**) DAB (**C**) and NBT (**D**) staining of csn-miR156f-2-5p-knockdown (AsODN) and sODN tea leaves subjected to 15% PEG at 0 h and 24 h. (**E**,**F**) Phenotype of damage (**E**) and *Fv/Fm* values (**F**) of csn-miR156f-2-5p-knockdown (AsODN) and sODN tea leaves subjected to 15% PEG after 0 h and 24 h that were used to assess drought stress resistance. Blue indicates a normal state of the photosynthetic apparatus, while green and yellow indicate damage to photosystem II due to drought. (**G**,**H**) Total chlorophyll (**G**) and proline content (**H**) of csn-miR156f-2-5p-knockdown (AsODN) and sODN tea leaves subjected to 15% PEG after 0 h and 24 h (* *p* < 0.05 and ** *p* < 0.01).

As there is no existing efficient and stable genetic transformation system for the tea plant, we used a gene-specific antisense oligodeoxynucleotide (AsODN) suppression strategy to knockdown csn-miR156f-2-5p in order to further confirm that csn-miR156f-2-5p participates in drought tolerance in tea plants. Drought stress usually causes osmotic stress in plants, which leads to restricted plant development [4]. Therefore, we intended to simulate drought with PEG 6000; firstly, we observed the expression pattern of csn-miR156f-2-5p during a 15% (*w*/*v*) polyethyleneglycol (PEG) treatment of tea plants. In contrast with previous natural drought results, csn-miR156f-2-5p increased significantly at 4 h and 12 h after PEG treatment and then decreased at 24 h (Figure S5). Subsequently, AsODN and sODN solutions were injected into the leaves of hydroponic tea plants, respectively. After 24 h of incubation after the injection of the AsODN solution, we successfully knocked down the expression of csn-miR156f-2-5p relative to the control (Figure 6A). The tea plants of the csn-miR156f-2-5p-knockdown (AsODN) and control (sODN) tea plants were immersed in a 15% PEG solution to assess drought tolerance. After 24 h of drought, the csn-miR156f-2-5p-knockdown tea plants showed a more severe degree of wilting than the controls (Figure 6B). Diaminobenzidine (DAB) staining (Figure 6C) and nitroblue tetrazolium (NBT) staining (Figure 6D) showed that more H_2_O_2_ and O_2_^−^ accumulated in the AsODN leaves than in the sODN leaves after 24 h of drought. Consistent with their phenotypes, chlorophyll fluorescence imaging demonstrated that the leaves of the csn-miR156f-2-5p-knockdown plants maintained a lower level of chlorophyll fluorescence (Figure 6E), and the *Fv/Fm* ratio was significantly decreased in the csn-miR156f-2-5p-knockdown tea plants compared with that of the controls (Figure 6F). In addition, the total chlorophyll content of the csn-miR156f-2-5p-knockdown plants was significantly lower than that of the control plants (Figure 6G); the proline content was significantly higher (Figure 6H), indicating that csn-miR156f-2-5p plays a role in tea plants’ response to drought.

### 2.7. Prediction and Expression Analysis of csn-miR156f-2-5p Target Genes in Tea Plants

**Figure 7:**
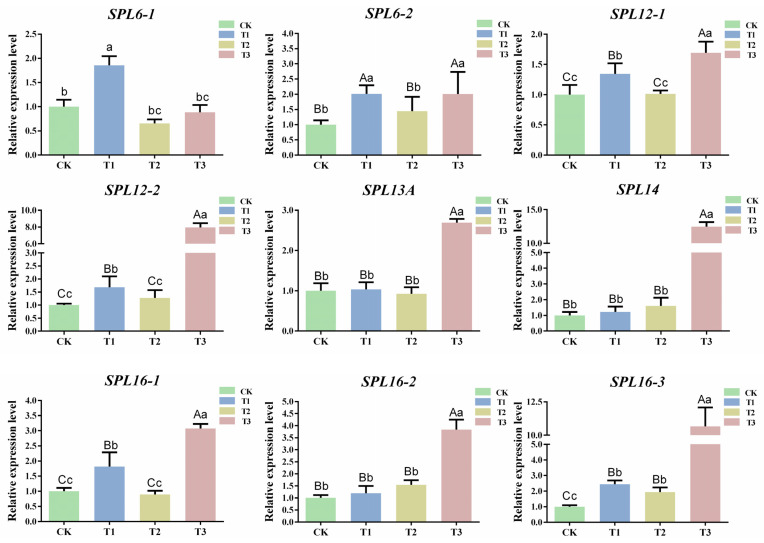
Expression patterns of targets during subjection to different degrees of drought. Data are the means of three independent replicates ± standard deviation (SD). Different lowercase letters indicate significant differences (*p* < 0.05); different uppercase letters indicate highly significant differences (*p* < 0.01).

To further explore the mechanism of csn-miR156f-2-5p in the drought resistance of tea plants, the target genes of csn-miR156f-2-5p were predicted using the psRNATarget website (https://www.zhaolab.org/psRNATarget/; accessed on 3 August 2023), and the results showed that nine SPL genes were targeted by csn-miR156f-2-5p (Table S2). These *SPL* genes include two *SPL6* (CsTGY06G0000542 and CsTGY15G0001546), two *SPL12* (CsTGY06G0000121 and CsTGY10G0000091), one *SPL13A* (CsTGY15G0000933), one *SPL14* (CsTGY10G0002410) and three *SPL16* (CsTGY02G0000059, CsTGY02G0001323, and CsTGY10G0000081) genes (Table S3). In order to further understand the expression patterns of *CsSPLs*, the expression patterns of *CsSPLs* in different tissue parts of the tea plants (buds, flowers, stems, young leaves, old leaves, and roots) were analyzed (Figure S6). The results showed that the expression levels of *SPL* genes varied in different kinds of tissue, mainly in the buds, stems, and leaves. In particular, the expression levels of CsTGY06G0000121 (*SPL12-1*) and CsTGY10G0002410 (*SPL14*) were higher in buds and leaves. Subsequently, the expression patterns of nine *SPL* genes were explored under different degrees of drought (Figure 7). The expression levels of five *CsSPL* target genes (*SPL6-2*, *SPL12-1*, *SPL12-2*, *SPL16-1*, and *SPL16-3*) showed a similar upward trend, all of which were significantly upregulated compared with CK at T1 and T3. The expression levels of *SPL6-1* and *SPL13A* increased significantly at T1. In addition, compared with CK, the expression levels of *SPL14* and *SPL16-2* continuously increased from T1 to T3. In most cases, miRNAs inhibited the expression of target genes. The results indicated that nine *CsSPL* genes were responding to drought stress, among which *SPL14* and *SPL16-2* were negatively correlated with the expression of csn-miR156f-2-5p during subjection to different degrees of drought stress.

### 2.8. Csn-miR156f-2-5p Suppresses the Expression of CsSPL14 in Tea Plants

**Figure 8:**
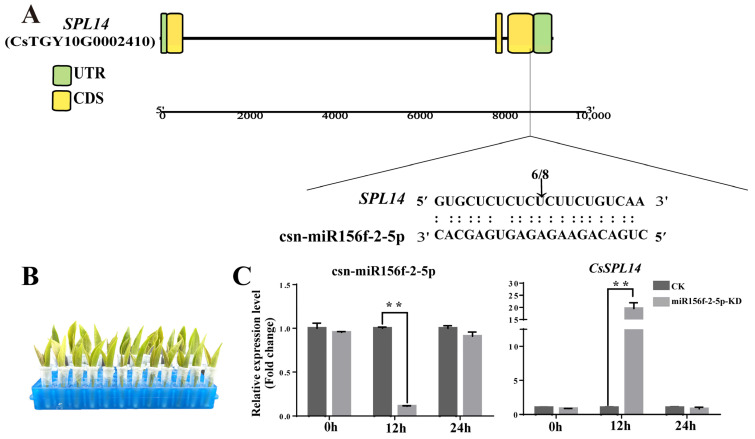
Verification of the relationship between csn-miR156f-2-5p and *CsSPL14*. (**A**) *CsSPL14* cleavage sites of csn-miR156f-2-5p, as identified using 5′RLM-RACE. (**B**) Knockdown of csn-miR156f-2-5p (miR156f-2-5p-KD) with AsODN and incubation, with a solution containing sense oligonucleotide (sODN) serving as a control. (**C**) RT-qPCR verification of csn-miR156f-2-5p knockdown (KD) and changes in the expression levels of *CsSPL14* after 0 h, 12 h, and 24 h of incubation (** *p* < 0.01).

Due to its low background expression level, only *CsSPL14* was successfully confirmed to contain cleaved sites. Multiple sequence alignment showed that the csn-miR156f-2-5p/*CsSPL14* duplex is cleaved between the 10th and 11th nucleotide from the 5′ end of csn-miR156f-2-5p (Figure 8A). These results confirmed that *CsSPL14* is the target gene of csn-miR156f-2-5p in tea plants. To explore the function of csn-miR156f-2-5p and its target gene *CsSPL14* in tea plants, we further verified the relationship between csn-miR156f-2-5p and *CsSPL14* expression. We incubated tender shoots of tea plants with either AsODN solution or sODN solution to knock down the expression of csn-miR156f-2-5p (Figure 8B). With the extension of incubation time, the expression level of csn-miR156f-2-5p decreased significantly in contrast to that of CK (sODN) at 24 h. The knockdown of csn-miR156f-2-5p (miR156f-2-5p-KD) expression led to increased levels of *CsSPL14* expression compared with those of CK at 24 h after incubation, suggesting that *CsSPL14* can be suppressed by csn-miR156f-2-5p (Figure 8C). These results illustrated that csn-miR156f-2-5p may play a role in the drought resistance mechanism of tea plants by inhibiting the expression levels of *CsSPL14*.

## 3. Discussion

### 3.1. Evolutionary Characteristics of the csn-miR156s Family

miR156s were some of the first miRNAs to be discovered; they are highly conserved [37] and widely distributed in 56 plant species, including angiosperms, gymnosperms, ferns, and bryophytes (Figure 1A). In recent years, with the rapid development of plant-genome-sequencing technology and the increasing abundance of plant genomes, research on miR156 in different plant species has gradually grown in prominence. For example, studies of the miR156 family in *M. truncatula*, *Glycine max*, *A. thaliana*, and *O. sativa* have shown that miR156s are an ancient miRNA family that play an important role in the regulation of plant growth, developmental stage transitions, flowering time, and stress responses [38,39,40,41,42]. Numerous studies have shown that the miR156 family is highly conserved in monocotyledons and dicotyledons [34]. In the present study, the evolutionary characteristics of the csn-miR156 family were analyzed in tea plants. In accordance with previous studies [14], our multiple sequence alignment results showed that miR156s were highly conserved in different species (Figure 1B). Sequences of csn-miR156s were relatively conserved among different plant species, but they also showed a degree of diversity. Studies have shown that the evolutionary distance of miRNA families is not closely related to the genetic relationship between species [33,37]. On the evolutionary tree, members of the csn-miR156 family of tea plants are distributed across various species (Figure 3), indicating that the csn-miR156 family of plants is highly complex in terms of its evolution; the evolution rates of each member of the csn-miR156 family are also different. This high level of complexity may explain why the evolutionary distance of the csn-miR156 gene family has little to do with how closely related species are to each other. Gene polymorphism involves gene replication, mutation, deletion, and duplication [43]. The mature sequences of csn-miR156 from the 5′ or 3′ arm of the precursors have a few base mutations, deletions, or insertions in addition to their most base-conserved sequences. We speculate that the diversity of the csn-miR156s family may also be a consequence of random mutations, which result in sequence diversity among csn-miR156s.

Based on small-RNA sequencing results and the NCBI database, the number of mature sequences of csn-miR156s was determined to be 47, while the number of corresponding precursors was 28. In the miRBase database, the quantities of mature sequences and precursors of *A. thaliana*, *M. truncatula*, and *O. sativa* were also inconsistent. The quantities of their mature sequences were 15, 15, and 18, respectively, while those of the precursors were 10, 10, and 12, respectively. The *CsMIR156s* were located on chr01, chr02, chr06, chr07, chr10, chr14, and chr15. Csn-miR156a/b/c/d/e/f were named according to the position of the mature sequence on their corresponding precursors (Figure 2B), while csn-miR156g/h/i/j/k did not correspond to the 28 precursor sequences. We observed that there was no one-to-one correspondence between the number of mature sequences and the number of precursor sequences in the same species; moreover, precursor genes may undergo mutations such as base substitution or deletion during the cutting and processing process to produce one or more mature sequences. It is also possible that some precursors were not detected due to the use of inadequate genome-sequencing techniques.

### 3.2. Different csn-miR156 Members May Respond to Drought Stress with Different Strategies

*Cis*-regulatory elements are important for studying the transcriptional regulation of genes. The prediction of *cis*-acting elements in the *CsMIR156s* promoter of the tea plant showed the existence of *cis* elements responsive to drought (MBS) (Figure 4). As expected, the expression patterns of csn-miR156s were affected by drought stress. The expression patterns of miR156 family members were different during subjection to drought stress, among which csn-miR156a-1-5p, csn-miR156a-4-3p, csn-miR156f-3-5p, and csn-miR156d-2-5p showed a pattern of upregulation during drought stress, while csn-miR156b-3-5p, csn-miR156b-7-5p, csn-miR156f-2-5p, csn-miR156f-4-5p, and csn-miR156f-4-3p showed a pattern of downregulation (Figure 5). It has been reported that different members of the same miRNA family may play various modal or regulatory roles in resistance to drought stress [44,45]. This may be due to the fact that the different csn-miR156 family members are derived from different precursors with different *cis*-responsive elements. These results indicate that members of the csn-miR156 family have a multitude of different roles in the drought resistance mechanism of tea plants, and the regulation of these plants’ genes during different degrees of drought may be complex and diverse. In fact, even within the same plant species, miRNAs can exhibit diverse responses to drought depending on specific conditions. For example, one study observed an increase in the expression level of miR398a/b in *M. truncatula* during drought stress [46], while another study reported a decrease in the expression level of this identical miRNA within the same plant species under similar drought conditions [47]. Indeed, the expression of csn-miR156f-2-5p in tea plants under PEG-simulated drought conditions was different from that found in a previous study. This difference may reflect different degrees of drought stress as well as the high sensitivity of some miRNAs to subtle differences in growth conditions. The differential expression of the same miRNA in the same plant species under drought conditions may be the result of different spatial–temporal behavior.

### 3.3. The csn-miR156 Family May Be Play a Role in the Drought Tolerance Mechanism by Cleaving CsSPLs

**Figure 9:**
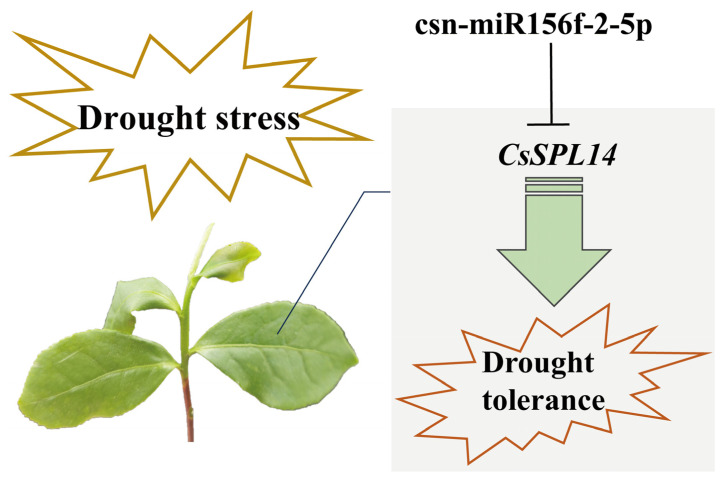
The potential drought tolerance mechanisms of csn-miR156f-2-5p-*CsSPL14* in tea plants.

In the present study, we successfully knocked down csn-miR156f-2-5p and found that tea plants with csn-miR156f-2-5p knocked down might be more sensitive to drought (Figure 6). This is consistent with previous findings showing that plants with miR156silence are more susceptible to drought stress than the corresponding WT with regard to *Arabidopsis* and apples [48,49].MicroRNAs function by cleaving the corresponding mRNA or inhibiting translation to regulate the expression of their target genes [12]. *SPLs* are genes with crucial roles in plants’ response to abiotic stress and their development, and they are the main target genes of miR156s [50]. Prediction of target genes revealed that csn-miR156f-2-5p targets *SPLs*, while RT-qPCR showed that *SPLs* respond to drought stress (Figure 7). This prediction was verified using 5′ RLM-RACE, which indicated that csn-miR156f-2-5p cleaved *CsSPL14* genes (Figure 8A). We also observed that the expression level of csn-miR156f-2-5p was inhibited by AsODN technology, and the expression level of *CsSPL14* was significantly increased (Figure 8B). The miR156ab-*SPL* module has been shown to improve drought resistance by accumulating auxin to maintain growth and by enhancing the activities of antioxidant enzymes in *Malus sieversii* [49]. Moreover, the drought tolerance of miR156-overexpressing and *SPL13*-silenced plants was significantly improved in alfalfa. Their survival rate and antioxidant enzyme activity were higher than those of the control [13]. In cassava, miR156-*MeSPL9* affected drought resistance by regulating the levels of protective metabolites and the jasmonic acid (JA) signaling pathway [51]. Therefore, we speculated that the cleaving of *CsSPL14* by csn-miR156f-2-5p could be an important mechanism involved in the mediation of drought resistance in tea plants (Figure 9).

Although it has been demonstrated that miR156-*SPL* modules are involved in plant responses to drought stresses, miR156-*SPL* combinations involving different *SPLs* may have different functions in different species. Therefore, it is necessary for us to further explore how the csn-miR156f-2-5p-*CsSPL14* module participates in the drought resistance mechanism of tea plants. In summary, our results demonstrate that the csn-miR156f-2-5p-*CsSPL14* module participates in drought tolerance in tea plants, providing a new research idea for improving tea plants’ drought resistance and productivity in terms of both yield and quality.

## 4. Materials and Methods

### 4.1. Plant Materials

The experimental materials were specimens of *C. sinensis* ‘Tieguanyin’. The experiment was conducted in the greenhouse of the College of Horticulture of Fujian Agriculture and Forestry University in June 2022 (119°14′ E, 26°05′ N). The daily temperature range of the greenhouse was 28–35 °C, and the atmospheric relative humidity was 50–75%. The soil field water capacity was 24%. Drought treatment was performed according to the method reported by Guo et al. [30]. The experimental design comprised treatment in four different forms: normal water supply (in which the soil water content was 80% ± 5% of the field water capacity; CK = 18.87%); mild drought stress treatment (wherein the soil water content was 60% ± 5% of field water capacity; T1 = 14.28%), moderate drought stress treatment (in which the soil water content was 40% ± 5% of the field water capacity; T2 = 9.96%); and severe drought stress (in which the soil water content was 20% ± 5% of the field water capacity; T3 = 5.90%). Each treatment was repeated 10 times. Tender *C. sinensis* leaves were fixed with liquid nitrogen and stored in a refrigerator at −80 °C until further analysis.

The relative water content of the soil was determined using the NY/T 52-1987 method. The relative water content of leaves was determined using the GB/T 8304-2002 method [45].

### 4.2. Bioinformatic Analyses of csn-miR156s and Target Gene Prediction

A total of 447 mature sequences of miR156 from 54 species were downloaded from miRbase, and we visualized the distribution of miR156 in 56 plant species using Origin (v2021) software. Some 47 mature sequences and 28 precursor sequences of csn-miR156s were obtained from the NCBI website (https://www.ncbi.nlm.nih.gov/; accessed on 18 June 2023) and named (Table S4). Subsequently, *P. patens*, *S. moellendorffii*, *O. Sativa*, *G. max*, *V. vinifera*, *A. thaliana*, *M. truncatula*, *N. tabacum*, *B. napus*, and *P. abies* members of the miR156 family were downloaded using the miRbase (Release 22.1) (http://www.mirbase.org/; accessed on 18 June 2023) database, which was used for subsequent analysis. DNAMAN 6.0 was used to analyze the csn-miR156 and pre-miR156 sequences. MEGA 10.0 software was used to construct a phylogenetic tree for the above sequences. The proximity neighbor-joining (NJ) method was implemented, and the Bootstrap coefficient was set to 1000. Evolution trees of csn-miR156s were visualized using Evolview (https://www.evolgenius.info/; accessed on 20 June 2023).

The secondary structure of csn-miR156 precursor sequences was predicted using RNAfold online website (http://rna.tbi.univie.ac.at/cgi-bin/RNAWebSuite/RNAfold.cgi; accessed on 25 July 2023). The 2000 bp upstream sequences of each csn-MIR156 and their *cis*-elements were predicted using the PlantCARE program (http://bioinformatics.psb.ugent.be/webtools/plantcare/html/; accessed on 28 July 2023).

Based on genome sequences of ‘Tieguanyin’ cultivars [52], the csn-miR156 targets were predicted using the psRNATarget (http://plantgrn.noble.org/psRNATarget/?function=3; accessed on 3 August 2023) online tool. The default parameters were applied, but the expected value was set to 2.0. 

### 4.3. Suppression of csn-miR156f-2-5p Expression in Tea Leaves Treated with AsODN

Candidate AsODNs were selected using Soligo online website (http://sfold.wadsworth.org/cgi-bin/soligo.pl; accessed on 4 August 2023), using csn-miR156f-2-5p as the input sequence (Table S5). To knock down csn-miR156f-2-5p expression, 1 mL of 50 μM AsODN-csn-miR156f-2-5p solution was injected into the leaves of hydroponic tea plant seedlings, and seedlings injected with sODN were used as controls. After incubation for 24 h, tea plants were treated with 15% (*w*/*v*) polyethyleneglycol (PEG) 6000 for 24 h (sODN/AsODN) [6]. Three independent biological replicates were established for each treatment. The obtained leaves were immediately frozen in liquid nitrogen and stored at 80 °C for further analysis.

Using Soligo online website (http://sfold.wadsworth.org/cgi-bin/soligo.pl; accessed on 4 August 2023), the antisense oligonucleotides for csn-miR156f-2-5p were designed, with csn-MIR156f-1 being used as an input sequence (Table S5). To knock down csn-miR156f-2-5p expression, one bud and two leaves of a freshly detached healthy plant of the variety ‘Tieguanyin’ were incubated in a 1.5 mL microcentrifuge tube that contained 50 μM of AsODN solution (containing 80 mM of sucrose solution) for various times (24 h and 48 h). Healthy tea branches incubated in sense ODNs (sODNs) together with 80 mM sucrose solution were used as controls [53]. The leaves were sampled at different time intervals to analyze gene expression levels. Three independent biological replicates were established for each treatment. The obtained leaves were immediately frozen in liquid nitrogen and stored at 80 °C for further analysis.

### 4.4. DAB and NBT Staining

Leaves were placed in a staining solution of 1 mg/mL of DAB or NBT (Solarbio, Beijing, China) prepared with 0.05 mol/L of sodium phosphate buffer (pH 7.5), evacuated for 5 min, and incubated in the dark at room temperature for 8 h before decolorization in 95% ethanol.

### 4.5. Determination of Fv/Fm, Chlorophyll, and Proline Content

The maximum efficiency of the photosystem II photochemistry (*Fv/Fm*) was measured using a chlorophyll fluorescence imaging system (IMAGING-PAM, Walz) after 30 min of dark adaptation and quantified using Origin (v2021) software. The chlorophyll content and proline content were determined using a chlorophyll content assay kit (HERUI, Fuzhou, China) and proline content kit (HERUI, Fuzhou, China), respectively.

### 4.6. RNA Extraction and RT-qPCR Analysis

Total RNA was extracted using Transzol UP (TransGen Biotech, Beijing, China). *CsSPL* genes and the first-strand cDNA of miRNA were synthesized using the TransScript^®^ Uni One-Step gDNA Removal, cDNA Synthesis SuperMix (TransGen, Beijing, China), and TranScript^®^ miRNA First-Strand cDNA Synthesis SuperMix (TransGen, Beijing, China) kits, respectively. The csn-miR156s, *CsSPLs*, and reference gene primers were designed using the Primer3 (https://bioinfo.ut.ee/primer3/; accessed on 5 August 2023) website (Table S5). RT-qPCR analysis of the csn-miR156s and their targets was conducted using TransStart^®^ Tip Green qPCR SuperMix (TransGen, Beijing, China) via an ABI StepOnePlus™ Real-Time PCR System (Applied Biosystems, Waltham, MA). CsmiR222 and *CsGAPDH* were used as internal controls for the csn-miR156s and *CsSPLs*, respectively. The 2^−ΔΔCt^ method was used to calculate relative expression. Finally, Tbtools and Prism 5 software were used to draw heat maps and bar charts.

### 4.7. Cleavage Site Identification with Modified 5′ RLM-RACE

Experimental validation of the predicted targets was conducted using a FirstChoice™ RLM-RACE Kit (Thermo Fisher Scientific, Carlsbad, CA, USA). The target gene cleavage primers were designed using DNAMAN 6.0 (Table S5). Two rounds of nested PCR amplification were performed, using the 5′ RACE outer primer and inner primer alongside target-gene-specific outer primer and inner primers, respectively. The PCR products were purified using an EasyPure^®^ Quick Gel Extraction Kit (TransGen, Beijing, China) and cloned into a Blunt Zero cloning vector (TransGen, Beijing, China); subsequently, single colonies of putative transformants of at least eight independent clones were sequenced.

### 4.8. Statistical Analysis

Data were analyzed using SPSS 25 software and are presented as the means ± SD of three independent biological replicates unless otherwise indicated. Significances were determined at *p* < 0.05 via one-way ANOVA analysis.

## 5. Conclusions

In this study, a total of 47 mature members and 28 precursor members of the miR156 family were identified in tea plants; they were found to be evolutionarily conservative and diverse. Moreover, our RT-qPCR analysis indicated that csn-miR156 family members are active in response to drought stress. In knocking down csn-miR156f-2-5p, we found that affected tea plants might be more sensitive to drought. The prediction and expression levels of target genes indicated that *CsSPLs* were involved in drought response. The 5′RACE and AsODN results demonstrated that csn-miR156f-2-5p cleaved and suppressed *CsSPL14*. Furthermore, AsODN confirmed that csn-miR156f-2-5p negatively regulated the expression of *CsSPL14*.

## Data Availability

Data is contained within the article or supplementary material.

## Supplementary Materials

The following supporting information can be downloaded at https://www.mdpi.com/article/10.3390/plants13020201/s1. Figure S1: Phylogenetic tree of plants’ miR156 sequences. vvi, *Vitis vinifera*; smo, *Selaginella moellendorffii*; osa, *Oryza sativa*; csn, *Camellia sinensis*; ath, *Arabidopsis thaliana*; gma, *Glycine max*; mtr, *Medicago truncatula*; nta, *Nicotiana tabacum*; bna, *Brassica napus*; pab, *Picea abies*; ppt, *Physcomitrella patens*. Figure S2: Sequence alignment analysis of miR156s. Different highlight colors represent different homology levels of sequence. Black, ≥100%; red, ≥75%; green, ≥50%. Figure S3: Sequence alignment among *C. sinensis* pre-miR156s. Different highlight colors represent different homology levels of sequence. Black, ≥100%; red, ≥75%; green, ≥50%. Figure S4: Stem–loop structures of csn-MIR156s in *C. sinensis*. The mature miRNA sequences are marked with a black line. Figure S5: Expression patterns of the csn-miR156f-2-5p at 0 h, 2 h, 4 h, 12 h, and 24 h after 15% PEG treatment. Data are the mean of three independent replicates ± standard deviation (SD). Different lowercase letters indicate significant differences (*p* < 0.05); different uppercase letters indicate highly significant differences (*p* < 0.01). Figure S6: Tissue-specific gene expression patterns of nine *SPL* genes targeted by csn-miR156f-2-5p in buds, flowers, stems, leaves, and roots. Table S1: *Cis*-Acting regulatory element analysis of MIR156 promoters. Table S2: Summary of target genes corresponding to csn-miR156f-2-5p. Table S3: Identification of target genes corresponding to csn-miR156f-2-5p. Table S4: Identification of 47 mature miRNA sequences and 28 precursor sequences. Table S5: The gene primers presented in this paper.
