# Supplementary material for: Identification and Validation of the miR156 Family Involved in Drought Responses and Tolerance in Tea Plants (Camellia sinensis (L.) O. Kuntze)
Source: Plants (Basel). 2024 Jan 11;13(2):201. doi: 10.3390/plants13020201 (PMC10819883; doi:10.3390/plants13020201)
Supplement: Supplementary file 1 [file plants-13-00201-s001.zip › Supplementary Material (Figure).pdf]

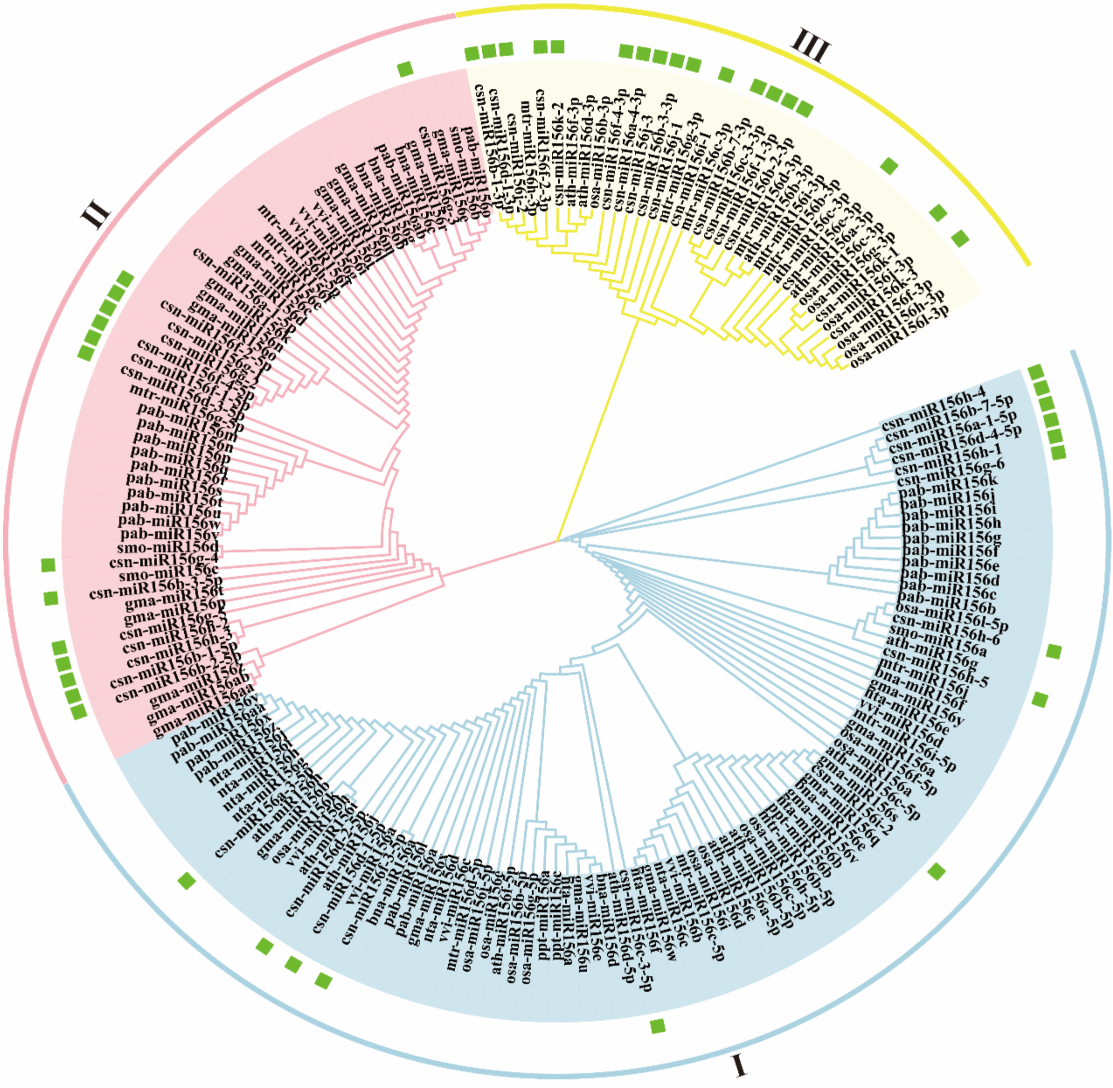

Figure S1. Phylogenetic tree of plants miR156 sequences. vvi. *Vitis vinifera*; smo. *Selaginella moellendorffii*; osa. *Oryza sativa*; csn. *Camellia sinensis*; ath. *Arabidopsis thaliana*; gma. *Glycine max*; mtr. *Medicago truncatula*; nta. *Nicotiana tabacum*; bna. *Brassica napus*; pab. *Picea abies*; ppt. *Physcomitrella patens*.

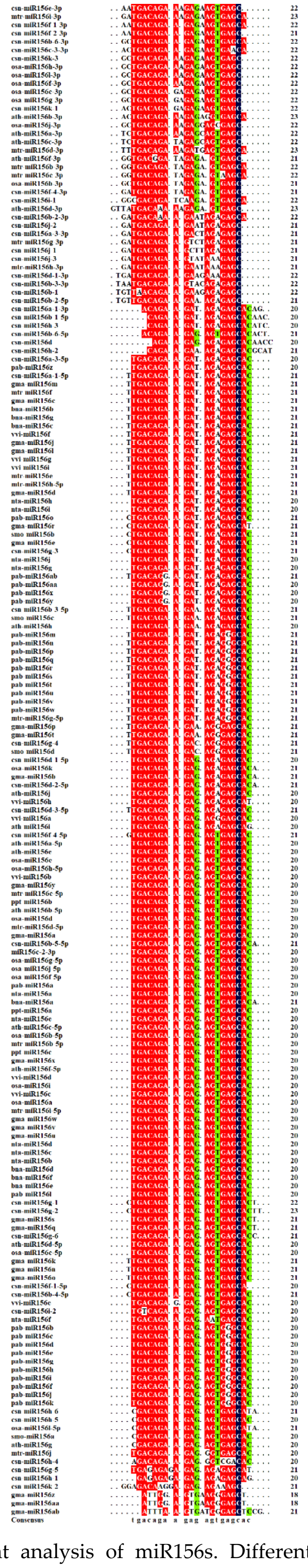

Figure S2. Sequence alignment analysis of miR156s. Different highlight colors represent different homology levels of sequence. Black. ≥100%; Red. ≥75%; Green. ≥50%.

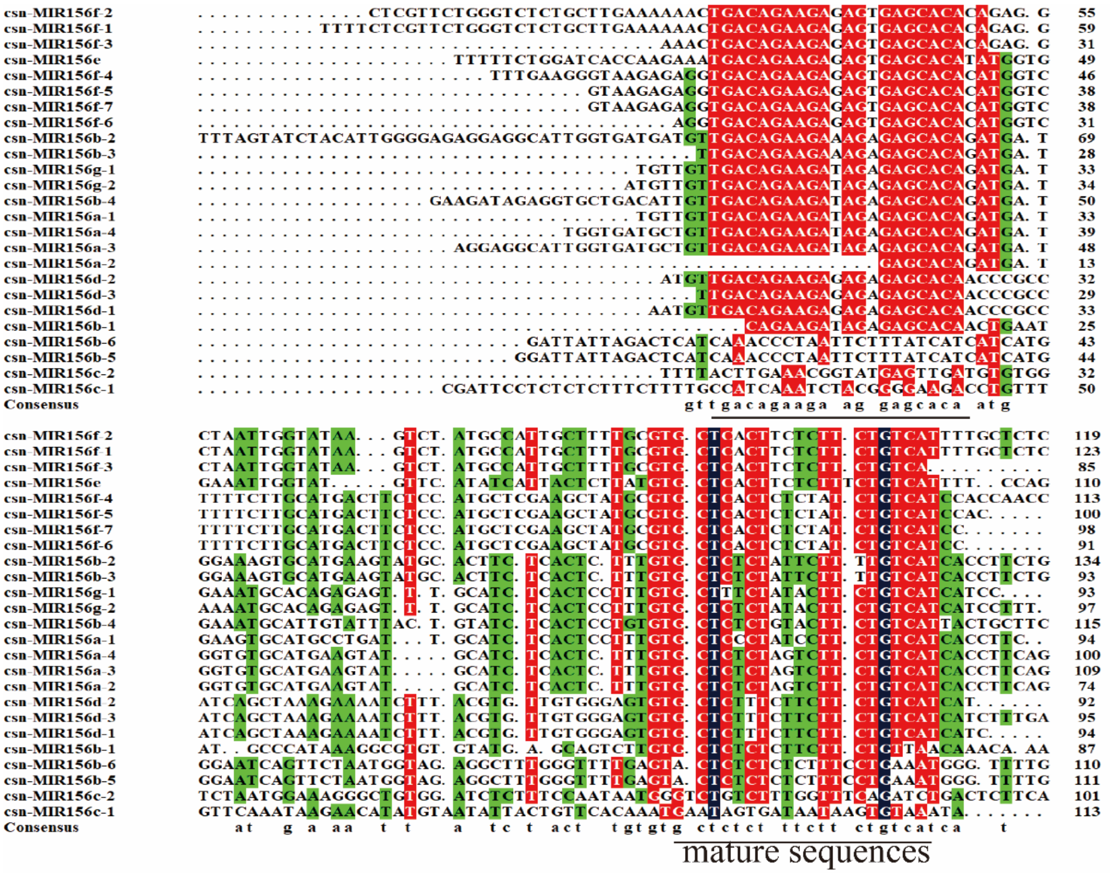

Figure S3. Sequences alignment among *C. sinensis* pre-miR156s. Different highlight colors represent different homology levels of sequence. Black. ≥100%; Red. ≥75%; Green. ≥50%.

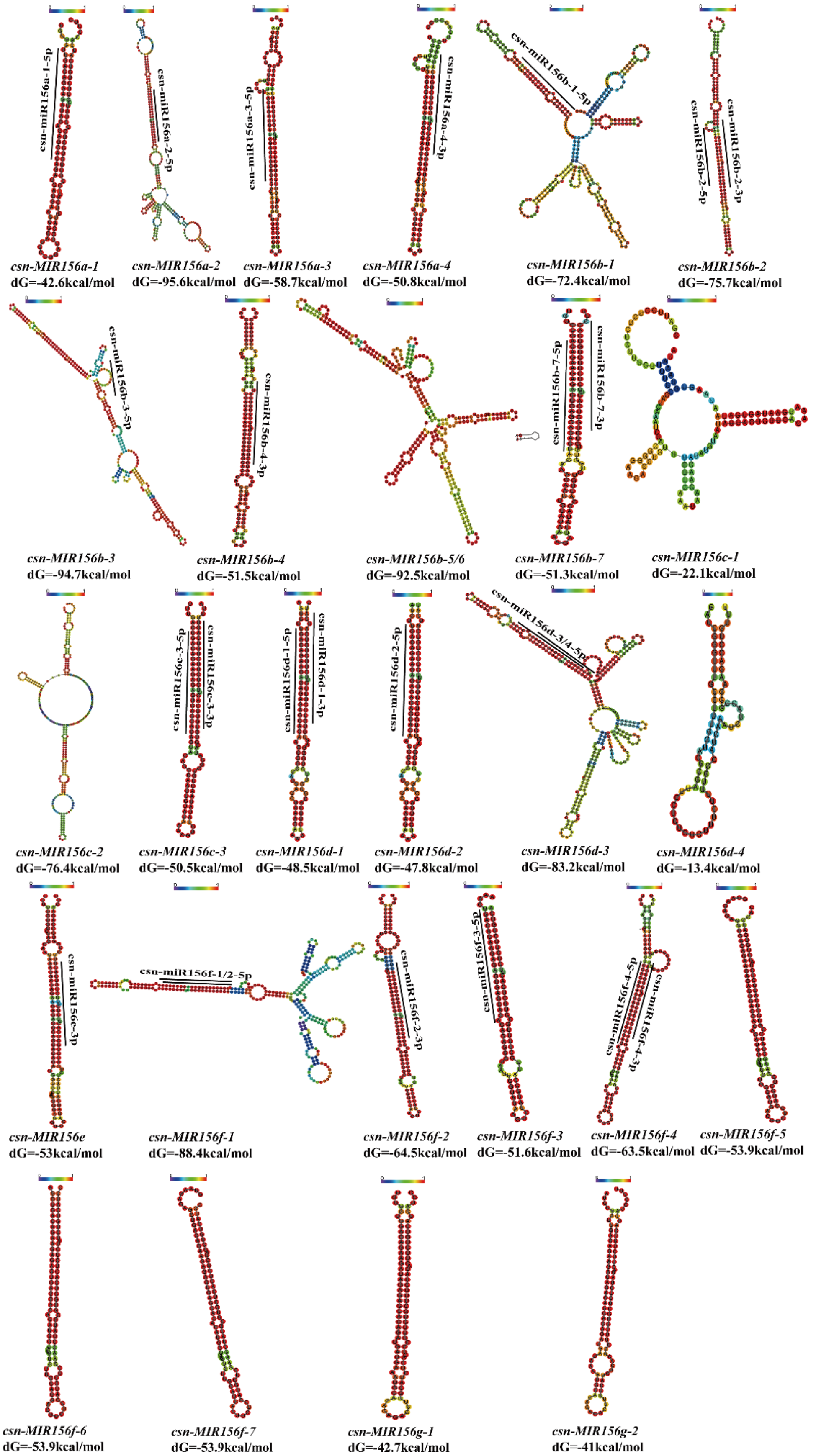

Figure S4. Stem-loop structures of csn-MIR156s in *C. sinensis*. The mature miRNA sequences are marked with a black line.

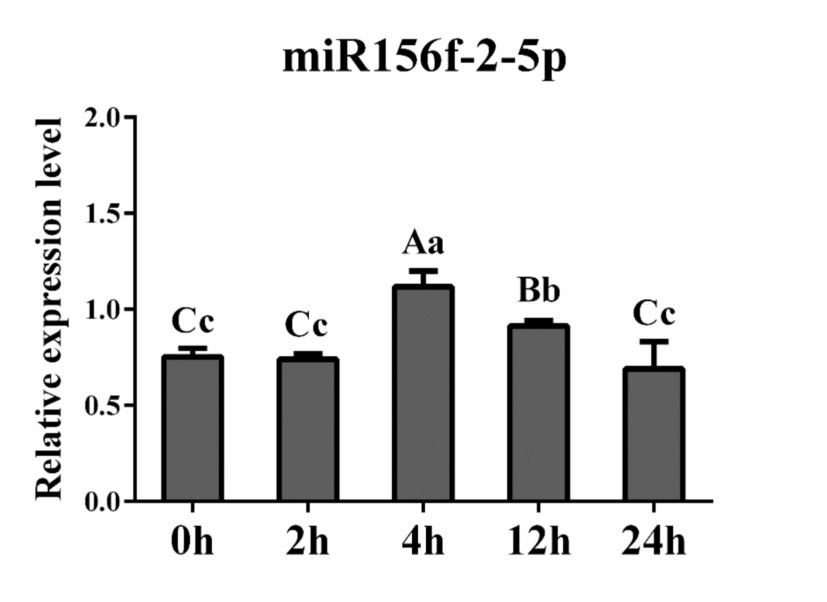

Figure S5. Expression patterns of the csn-miR156f-2-5p at 0 h, 2 h, 4 h, 12 h, and 24 h after 15% PEG treatment. Data were the mean of three independent replicates ± standard deviation (SD). Different lowercase letters indicate significant differences ($p < 0.05$); different uppercase letters indicate highly significant differences ($p < 0.01$).

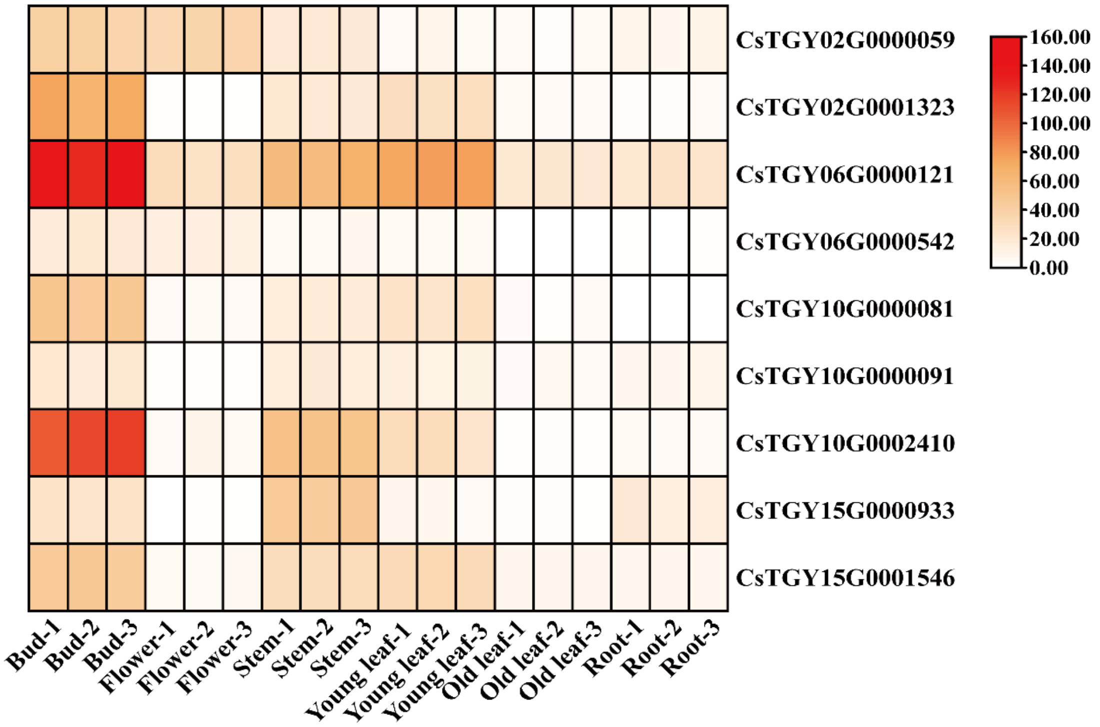

Figure S6. Tissue-specific gene expression patterns of nine *SPL* genes targeted by csn-miR156f-2-5p in bud, flower, stem, leaf, and root.
